# Development of the BWAT-CUA Scale to Assess Wounds in Patients with Calciphylaxis

**DOI:** 10.3390/diagnostics11040730

**Published:** 2021-04-20

**Authors:** Lisa J. Gould, Thomas E. Serena, Smeeta Sinha

**Affiliations:** 1 South Shore Health Center for Wound Healing, Weymouth, MA 02189, USA; 2 Serena Group Research Foundation, Cambridge, MA 02140, USA; serena@serenagroups.com; 3 Manchester Academic Health Science Centre, University of Manchester, Salford Royal NHS Foundation Trust, Stott Lane, Salford M6 8HD, UK; smeeta.sinha@srft.nhs.uk

**Keywords:** calciphylaxis, scale, rating, wound, healing

## Abstract

Calcific uremic arteriolopathy (CUA; calciphylaxis) is a severe form of vascular calcification with no approved therapies. A standardized wound assessment tool is needed to evaluate changes in calciphylaxis wounds over time. A prospective, single-arm study of 14 patients with calciphylaxis reported improvement for the primary endpoint of wound healing using the 13-item Bates-Jensen Wound Assessment Tool (BWAT), although that tool was developed for assessment of pressure ulcers. This report describes development of BWAT-CUA, an 8-item modification of BWAT focusing on prototypical features of calciphylaxis lesions. The BWAT-CUA has a range of 8 (best) to 40 (worst) and was used ad hoc to analyze BWAT data collected in the prospective study. Using BWAT-CUA, relative improvement in calciphylaxis wounds was 30% overall (from 21.2 to 14.9; *p* = 0.0016) and 34% in the subset of 12 patients with ulceration at baseline (from 23.3 to 15.3; *p* = 0.0002). BWAT-CUA is a primary endpoint in an ongoing randomized, placebo-controlled phase 3 study of SNF472 recruiting patients with end-stage kidney disease and at least one ulcerated calciphylaxis lesion. BWAT-CUA, a newly developed tool for assessment of calciphylaxis wound severity and improvements over time, may be used in clinical research and in clinical practice.

## 1. Introduction

Calcific uremic arteriolopathy (CUA or calciphylaxis) is a rare disease seen predominantly in patients with end-stage kidney disease (ESKD) on dialysis [1,2]. The reported prevalence of calciphylaxis among patients on dialysis is usually less than 1% [3,4,5,6,7], but the number of patients diagnosed with calciphylaxis is increasing due to both the increased prevalence of ESKD [8] and the increased recognition of calciphylaxis [6]. The true incidence of calciphylaxis may be higher than reported, because it is not always recognized or diagnosed.

Risk factors for development of calciphylaxis in patients on dialysis include diabetes, obesity, female sex, white race, selected treatments (vitamin D, cinacalcet, and warfarin), and higher serum levels of calcium, phosphorus, and parathyroid hormone [9]. The pathophysiology of calciphylaxis includes deposition of calcium in the tunica media of smaller arteriolar vessels, local inflammation, and arteriolar thrombosis, leading to regional ischemia and subsequent necrosis of subcutaneous fat and the overlying skin [1].

**Figure 1:**
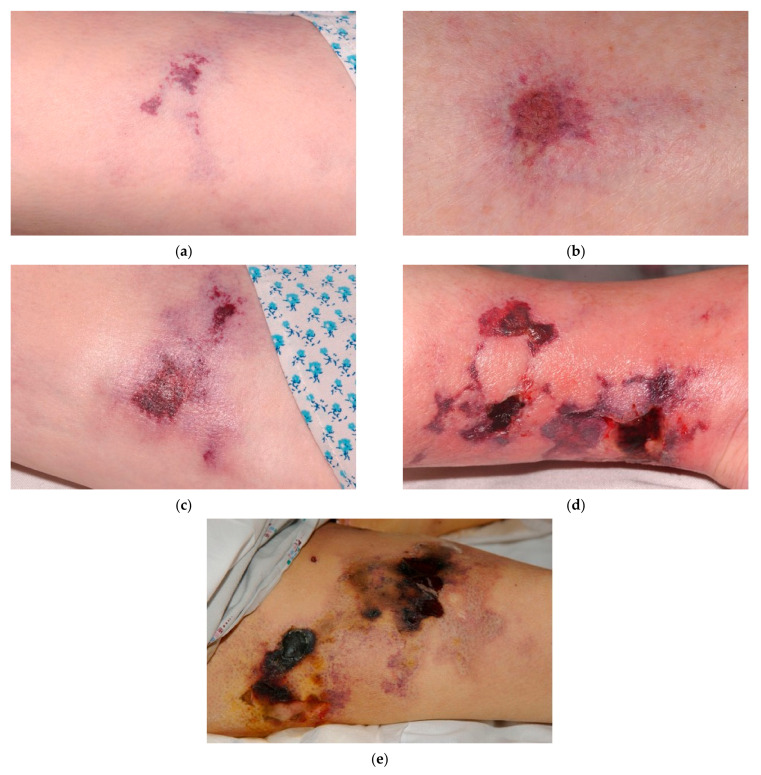
Examples of typical calciphylaxis wound presentations. As shown in the images, milder calciphylaxis wounds (**a**–**c**) typically have color changes in the surrounding skin and relatively low exudate, without evidence of infection. More severe, advanced calciphylaxis wounds (**d**,**e**) develop ulceration and necrotic tissue with eschar obscuring the actual depth of the wound, as well as edema and erythema suggesting inflammation. When palpated, there is induration adjacent to the necrotic tissue in the more severe wounds.

The primary manifestations of calciphylaxis are lesions of the skin and subcutaneous tissues, most commonly in the truncal area and lower limbs [1,2]. Common characteristics of calciphylaxis lesions at diagnosis include tissue necrosis or full-thickness open wounds; induration is also commonly seen. Lesions and surrounding necrotic areas often show a characteristic plaque-like hardening. Typical characteristics for calciphylaxis lesions at presentation are shown for wounds of varying severity in Figure 1.

These progressive, ischemic ulcers are extremely painful, and patients often require opioids for pain control [10]. Patients with calciphylaxis also have increased susceptibility to wound infections, which are ultimately responsible for approximately 50% of mortality associated with calciphylaxis [11,12]. Calciphylaxis in patients with ESKD can be a life-threatening condition, with 1-year mortality rates exceeding 45% [9,11,13,14], and calciphylaxis independently increases the risk of death by up to 8-fold [5,6,14].

It is well accepted that acute wound healing depends on a brief influx of neutrophils and macrophages during the inflammatory phase [15]. In addition to clearing debris, these inflammatory cells secrete chemotactic factors that are crucial for progression to the proliferative phase. For optimal healing, the inflammatory phase must be limited in both magnitude and duration. In contrast, the majority of calciphylaxis lesions are highly inflammatory chronic wounds, with a local ischemic component that inhibits the progression from the inflammatory to the proliferative phase. Given the severity of the ulcers and the co-morbidities that are common in patients with calciphylaxis, complete wound healing may be delayed and complicated by superinfection, sepsis, and death [14,16,17].

Major challenges in the management of calciphylaxis include difficulty achieving pain control, poor wound healing, the lack of treatment options with demonstrated benefit, and the need for standardized methods to diagnose and monitor the progression of the characteristic wounds. Observational studies have shown that a minority of patients with calciphylaxis wounds achieve complete healing with current standard care [14,16,17]. Clinical care of patients with calciphylaxis includes topical wound care with measures to prevent infection, pain management, and various measures that are designed to reduce calcium deposition indirectly. To date, no therapeutic interventions including parathyroidectomy, altering dialysis regimen, off-label use of sodium thiosulfate or treatment with hyperbaric oxygen have been shown to improve wound healing in any published, randomized, controlled clinical trials [2,14,18,19,20,21,22,23,24].

To our knowledge, only one prospective clinical study has been published that assessed the efficacy and tolerability of a treatment for calciphylaxis in improving wound healing and pain. In this single-arm study, 14 patients with calciphylaxis were treated with SNF472, an investigational hydroxyapatite crystallization inhibitor, during thrice weekly hemodialysis sessions for 12 weeks [25]. The study met its primary endpoint, showing improvement in wound healing, as well as secondary endpoints including improvement in pain and wound-related quality of life.

## 2. BWAT: A Chronic Wound Assessment Tool

When the phase 2 study of SNF472 in patients with calciphylaxis was planned, a variety of tools for wound assessment were available, but very few had undergone rigorous validation. No tool was developed or validated specifically for calciphylaxis wounds and no prior study in calciphylaxis had evaluated wound healing with a dedicated wound assessment instrument. The Bates-Jensen Wound Assessment Tool (BWAT), which was selected to measure the primary study endpoint of calciphylaxis wound healing in the phase 2 study [25], is widely used for measuring and predicting wound healing in clinical settings and has been used successfully in randomized clinical studies for treatment of chronic wounds, including pressure ulcers, diabetic ulcers, and venous leg ulcers [26,27,28,29]. Thus, BWAT was selected as the primary efficacy measure in the phase 2 study to provide a well-accepted, objective, quantitative tool that could be applied systematically across study sites to assess changes in lesions over time.

The BWAT was originally developed as the Pressure Sore Status Tool (PSST); reliability and validity of the PSST/BWAT were demonstrated for pressure ulcers, and content validity was determined for leg ulcers and pressure ulcer healing [30,31,32]. The full version of BWAT includes 13 items, each scored from either 0 or 1 (best) to 5 (worst), and total BWAT scores range from 9 to 65 [33]. The 13 items address the following typical characteristics of leg ulcers and pressure ulcers: size, depth, edges, undermining, necrotic tissue type, necrotic tissue amount, exudate type, exudate amount, skin color surrounding the wound, peripheral tissue edema, peripheral tissue induration, granulation tissue, and epithelialization (Table 1).

The BWAT was not initially developed to evaluate wound healing over time; however, a few groups have investigated the use of selected items from the BWAT to measure changes over time for ulcerated wounds. Although it was not specifically validated as an independent assessment, a 5-item BWAT inflammation subscore was responsive to an intervention that promoted healing of pressure ulcers and venous leg ulcers [26]. Another clinical study selected eight items from the BWAT to assess diabetic foot ulcers; however, justification of that selection was not described [34].

## 3. Development of the BWAT-CUA Scale

To optimize the scale for evaluation of changes specific to calciphylaxis wounds, a group of clinician-researchers with expertise in calciphylaxis and wound healing collaborated to develop a modified version of the tool focusing on eight prototypical and clinically relevant features of calciphylaxis at diagnosis and during healing (Table 1) [1,2].

### 3.1. Necrotic Tissue Type and Necrotic Tissue Amount

Tissue necrosis is a common feature of calciphylaxis wounds. The presence and amount of necrotic tissue can indicate the severity of the disease. Calciphylaxis is usually diagnosed late in the pathological process, by which point many lesions already have necrotic features. The resolution of necrosis is a valuable diagnostic feature and a marker of progression/improvement.

### 3.2. Exudate Type and Exudate Amount

As part of the normal healing process, exudates are produced and are responsible for maintaining a moist wound bed necessary for healing. The inclusion of exudate type and amount as items of the BWAT is particularly pertinent to calciphylaxis wounds because an increase in exudate volume or viscosity may indicate an underlying infection, which is highly relevant to these wounds and related outcomes. Exudate is not recorded in all wound assessment tools and is particularly challenging in wound assessments that are dependent on visual images/photos. BWAT has the advantage of capturing this element.

### 3.3. Skin Color Surrounding Wound, Peripheral Tissue Edema, and Peripheral Tissue Induration

It is important to assess the surrounding skin as it may provide the first indication of impending further tissue damage, and induration or erythema may indicate infection. Edema, induration, and changes in the skin color surrounding the wound (erythema, suggesting increased blood flow to the area, or ecchymosis, suggesting ischemic changes) are often seen in calciphylaxis wounds. These features assist with the diagnostic process and are also important for monitoring of wound progression and presence of infection.

### 3.4. Granulation Tissue

The presence of granulation tissue is an important positive feature in wounds as it indicates commencement of healing, particularly in slow-healing wounds such as calciphylaxis, where changes in size and epithelialization may not be apparent for an extended period of time.

### 3.5. Items Excluded

We excluded 5 items of the BWAT from BWAT-CUA either because they are characteristic of pressure ulcers but not calciphylaxis wounds (undermining), or because they were not expected to be sensitive measures of improvement in slow-healing calciphylaxis wounds, (size, depth, edges, and epithelialization). Importantly, the BWAT size categories are very broad, reducing the sensitivity of the tool. According to the BWAT size categories, large improvements in wound size can occur without changing the BWAT size rating. For example, a wound that started at 16 cm^2^ and decreased to 4.1 cm^2^, representing more than a 70% improvement, would continue to have an unchanged BWAT size rating of 2. Thus, we recommend measuring the size of calciphylaxis wounds separately, as a continuous variable. The depth item was excluded as it was likely to be a binary measure for many calciphylaxis wounds (4 = obscured by necrosis or 5 = full thickness skin loss), making it redundant with the BWAT item for necrotic tissue type.

## 4. Application of the BWAT-CUA Scale

In the phase 2 study of SNF472 in patients with calciphylaxis, investigators recorded all 13 items of the BWAT both prospectively at baseline and after 12 weeks of SNF472 treatment. In the primary report from that study, mean ± SD scores for total BWAT improved from 33.6 ± 9.2 at baseline to 25.6 ± 7.6 at week 12, with a statistically significant mean ± SD change of −8.1 ± 8.5 (95% CI: −12.7, −3.4; *p* < 0.001) for the primary study endpoint [25]. There was also qualitative improvement in wound healing for 7 of 9 patients with open ulcers at baseline, as assessed by two external experts who reviewed photographs from each visit.

**Figure 2:**
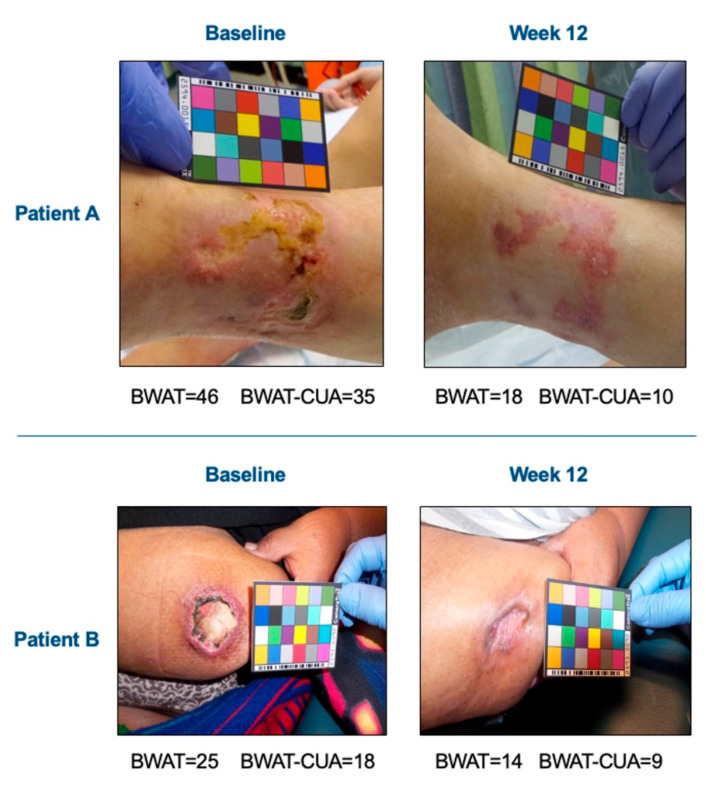
BWAT-CUA scores versus total BWAT scores in a clinical study. Images were obtained before and after 12 weeks of open-label treatment with the hydroxyapatite crystallization inhibitor, SNF472. Investigators rated all 13 items of the BWAT prospectively and the total BWAT score was calculated for the primary efficacy endpoint of calciphylaxis wound healing. BWAT-CUA scores were calculated ad hoc from the eight items selected for this tool.

Sample scores using the original BWAT or the BWAT-CUA are shown in Figure 2 for two patients with qualitative healing in that study. Using the prospectively rated scores for each BWAT item, relative improvements for one patient were 61% for total BWAT (from 46 to 18) and 71% for BWAT-CUA (from 35 to 10), while for the other patient they were 44% for total BWAT (from 25 to 14) and 50% for BWAT-CUA (from 18 to 9). Thus, the BWAT-CUA score appeared to be more sensitive to improvement than the total BWAT score in these patients.

**Figure 3:**
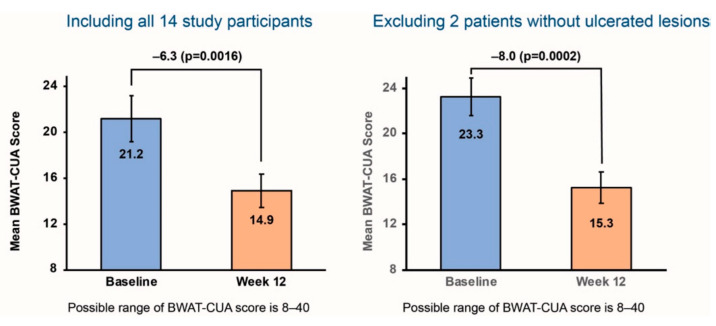
Ad hoc analysis of BWAT-CUA scores before and after 12 weeks of SNF472 treatment in a clinical study. The **left panel** in each image shows BWAT-CUA scores for all 14 study participants. The **right panel** shows BWAT-CUA scores excluding 2 patients without open ulcers at baseline.

By applying the same method across all 14 patients in that study (Figure 3), the relative improvement in mean BWAT-CUA scores was 30%, from 21.2 at baseline to 14.9 at the end of treatment (*p* = 0.0016).

## 5. Future Directions

As understanding increases about the pathophysiology of calciphylaxis and other forms of vascular calcification, a standardized approach for the evaluation of calciphylaxis wounds will be useful to ensure efficacy can be compared across different studies and different investigational approaches. Based on the results from the phase 2 study of SNF472, patients with calciphylaxis and ESKD are being recruited in CALCIPHYX, a multinational, randomized, placebo-controlled, phase 3 study of SNF472 (NCT04195906). This study is using BWAT-CUA to assess improvements in wound healing with 12 weeks of SNF472 treatment (versus placebo) as an alternate primary study endpoint; the other primary endpoint is change in pain. The BWAT-CUA was developed post-hoc from wound images. The CALCIPHYX study will use high resolution photographs with the patient in the same position each time. In addition to still photos, a video is taken of the dressing removal and wound evaluation. This allows expert review of the BWAT-CUA elements remotely. To enroll in the study, patients must have at least one calciphylaxis lesion with ulceration of the epithelial surface. In the phase 2 study, 2 of 14 patients did not have ulceration at study entry [25]. However, BWAT was developed as a tool to evaluate open wounds; thus, no improvement or minimal improvement could be observed in these subjects. When we excluded those two patients from the ad hoc analysis, the mean BWAT-CUA score was higher than in the total study population (Figure 3). The absolute improvement (−8.0) and relative improvement (34%) for mean BWAT-CUA scores from baseline to end of treatment (from 23.3 to 15.3; *p* = 0.0002) were also larger in this subgroup. Thus, we expect that the BWAT-CUA will be a tool that is both sensitive and specific to assess improvement in wound healing with SNF472 versus placebo within the patient population being recruited for the phase 3 study, and that it could also be used in other prospective studies of investigational treatments for calciphylaxis.

The BWAT-CUA may also have utility for clinical practice, with proper training. Studies have shown that a brief training session (30 min) can improve the interrater reliability coefficient for BWAT total score to greater than 0.95 [30,35].

## 6. Conclusions

Facing both the need for a wound assessment instrument for a calciphylaxis study and no prior successful efforts in therapeutic development or a validated tool, we identified BWAT as the most appropriate instrument for modification. Starting with the established 13-item BWAT tool for assessment of ulcers, we identified the eight items that represent prototypical features of calciphylaxis to create the BWAT-CUA. Our ad hoc analysis of prospectively collected BWAT scores before and after investigational treatment in a single-arm study showed improvement in BWAT-CUA scores, with greater sensitivity among patients with ulcerated calciphylaxis lesions. BWAT-CUA is being used for a primary study endpoint in a randomized, placebo-controlled trial of SNF472 that is now recruiting patients with ESKD and at least one ulcerated calciphylaxis lesion. The use of a standardized tool to assess these difficult wounds is necessary not only in clinical trials, but also to pursue a unified approach for evaluation of calciphylaxis lesions in clinical practice.

## Figures and Tables

**Table 1:** Item selection from the original Bates-Jensen Wound Assessment Tool (BWAT) to develop a modified version for calcific uremic arteriolopathy (BWAT-CUA).

BWAT Item	Scores	Included/Excluded in BWAT-CUA: Rationale
Necrotic tissue type	1 = None visible; 2 = White/grey non-viable tissue and/or non-adherent yellow slough; 3 = Loosely adherent yellow slough; 4 = Adherent soft black eschar; 5 = Firmly adherent hard black eschar	**Included** Calciphylaxis is often diagnosed late, when necrosis is already present. Reduction of necrotic tissue may be a sensitive indicator of improvement.
Necrotic tissue amount	1 = None visible; 2 = <25% of wound bed covered; 3 = 25% to 50% of wound covered; 4 = >50% but <75% of wound covered; 5 = 75% to 100% of wound covered	**Included** See necrotic tissue type.
Exudate type	1 = None; 2 = Bloody; 3 = Serosanguineous: thin, watery, pale red/pink; 4 = Serous: thin, watery, clear; 5 = Purulent: thin or thick, opaque, tan/yellow, with or without odor	**Included** Particularly pertinent in calciphylaxis wounds, which have a high risk of infection; ~50% of patients with calciphylaxis have sepsis as an attributable cause of death.
Exudate amount	1 = None, dry wound; 2 = Scant, wound moist but no observable exudate; 3 = Small; 4 = Moderate; 5 = Large	**Included** See exudate type.
Skin color surrounding wound	1 = Pink or normal for ethnic group; 2 = Bright red and/or blanches to touch; 3 = White or grey pallor or hypopigmented; 4 = Dark red or purple and/or non-blanchable; 5 = Black or hyperpigmented	**Included** Erythema is often seen in calciphylaxis wounds; it can assist with the diagnostic process as well as with monitoring wound progression and infection.
Peripheral tissue edema	1 = No swelling or edema; 2 = Non-pitting edema extends <4 cm around wound; 3 = Non-pitting edema extends >4 cm around wound; 4 = Pitting edema extends <4 cm around wound; 5 = Crepitus and/or pitting edema extends >4 cm around wound	**Included** Edema is often seen in calciphylaxis wounds; it can assist with the diagnostic process as well as with monitoring wound progression and infection.
Peripheral tissue induration	1 = None present; 2 = Induration <2 cm in any area around wound; 3 = Induration 2–4 cm extending <50% around wound; 4 = Induration 2–4 cm extending >50% around wound; 5 = Induration >4 cm in any area around wound	**Included** Induration is often seen in calciphylaxis wounds; it can assist with the diagnostic process as well as with monitoring wound progression and infection.
Granulation tissue	1 = Skin intact or partial thickness wound; 2 = Bright, beefy red; 75% to 100% of wound filled and/or tissue overgrowth; 3 = Bright, beefy red; >25% to <75% of wound filled; 4 = Pink, and/or dull, dusky red and/or fills <25% of wound; 5 = No granulation tissue present	**Included** As calciphylaxis wounds improve it is expected that there will be increased granulation tissue. Granulation tissue indicates commencement of healing, particularly for slow-healing calciphylaxis wounds.
**Excluded Items**		
Undermining	0 = Healed, resolved wound; 1 = None; 2 = Undermining <2 cm in any area; 3 = Undermining 2–4 cm involving <50% wound margins; 4 = Undermining 2–4 cm involving >50% wound margins; 5 = Undermining >4 cm or tunneling in any area	**Excluded** Undermining is not a characteristic feature of calciphylaxis wounds.
Size	0 = Healed, resolved wound; 1 = Length × width <4 cm^2^; 2 = Length × width 4 to <16 cm^2^; 3 = Length × width 16.1 to <36 cm^2^; 4 = Length × width 36.1 to <80 cm^2^; 5 = Length × width >80 cm^2^	**Excluded** Ranges are broad and wound size is not a sensitive measure for slow-healing wounds like calciphylaxis.
Depth	0 = Healed, resolved wound; 1 = Non-blanchable erythema on intact skin; 2 = Partial thickness skin loss involving epidermis and/or dermis; 3 = Full thickness skin loss involving damage or necrosis of subcutaneous tissue; may extend down to but not through underlying fascia; and/or mixed partial & full thickness and/or tissue layers obscured by granulation tissue; 4 = Obscured by necrosis; 5 = Full thickness skin loss with extensive destruction, tissue necrosis or damage to muscle, bone or supporting structures	**Excluded** The depth descriptions for BWAT correspond to pressure ulcer stages. Calciphylaxis lesions usually present as either necrotic or full-thickness lesions; thus, this item was most likely to be binary (4 or 5) and redundant with the BWAT item for necrotic tissue type.
Edges	0 = Healed, resolved wound; 1 = Indistinct, diffuse, none clearly visible; 2 = Distinct, outline clearly visible, attached, even with wound base; 3 = Well-defined, not attached to wound base; 4 = Well-defined, not attached to base, rolled under, thickened; 5 = Well-defined, fibrotic, scarred or hyperkeratotic	**Excluded** Edges are less relevant in slow-healing calciphylaxis wounds, which tend to heal from the base up.
Epithelialization	1 = 100% wound covered, surface intact; 2 = 75% to <100% wound covered and/or epithelial tissue extends >0.5 cm into wound bed; 3 = 50% to <75% wound covered and/or epithelial tissue extends to <0.5 cm into wound bed; 4 = 25% to <50% wound covered; 5 = <25% wound covered	**Excluded** Late feature of wound healing.
